# Supplementary material for: Non-coding RNA mediates the defense-associated reverse transcriptase (DRT) anti-phage oligomerization transition
Source: EMBO J. 2025 Aug 20;44(19):5429–42. doi: 10.1038/s44318-025-00544-8 (PMC12489045; doi:10.1038/s44318-025-00544-8)
Supplement: Supplementary file 1 — Appendix [file 44318_2025_544_MOESM1_ESM.pdf]

Appendix for

Non-coding RNA mediates the Defense-Associated Reverse Transcriptase anti-phage oligomerization transition

Jie Han[1,2,3#], Bin Liu[1,2#], Jingjing Tang[4,5#], Shuqin Zhang[1,2#], Xiaoshen Wang[1], Xuzichao Li[6], Qian Zhang[1], Zhikun Liu[1], Wanyao Wang[7], Yingcan Liu[1], Ruimin Zhou[1], Hang Yin[1], Yong Wei[6], Zhuang Li[8], Minjie Zhang[7]*, Zengqin Deng[4]*, Heng Zhang[1]*

1. Key Laboratory of Immune Microenvironment and Disease (Ministry of Education), State Key Laboratory of Experimental Hematology, Tianjin Medical University Cancer Institute and Hospital, The Province and Ministry Co-sponsored Collaborative Innovation Center for Medical Epigenetics, Tianjin Institute of Immunology, School of Basic Medical Sciences, Tianjin Medical University, Tianjin 300070, China.
2. Tianjin Key Laboratory of Cellular Homeostasis and Disease, Department of Biochemistry and Molecular Biology, Tianjin Medical University, Tianjin 300070, China
3. Department of Anatomy, School of Basic Medical Sciences, Tianjin Medical University, Tianjin 300070, China.
4. Key Laboratory of Virology and Biosafety, Wuhan Institute of Virology, Chinese Academy of Sciences, Wuhan, China.
5. University of Chinese Academy of Sciences, Beijing, China.
6. The Cancer Hospital of the University of Chinese Academy of Sciences (Zhejiang Cancer Hospital), Institute of Basic Medicine and Cancer (IBMC), Chinese Academy of Sciences, Hangzhou, China.
7. Department of Bioinformatics, School of Basic Medical Sciences, Tianjin Medical University, Tianjin 300070, China.
8. State Key Laboratory of Biocatalysis and Enzyme Engineering, School of Life Sciences, Hubei University, Wuhan 430062, China
# The four authors contribute equally to this work.
*Correspondence: zhangmj@tmu.edu.cn (M.Z.); dengzengqin@wh.iov.cn (Z.D.); zhangheng134@gmail.com (H.Z.)

Table of Contents

Appendix Figure S1......3
Appendix Figure S2......4
Appendix Figure S3......5
Appendix Figure S4......6
Appendix Figure S5......7
Appendix Figure S6......8
Appendix Figure S7......9
Appendix Figure S8......10
Appendix Figure S9......11
Appendix Figure S10......12

Appendix Figure S11...13
Appendix Figure S12...14
Appendix Table S1 15

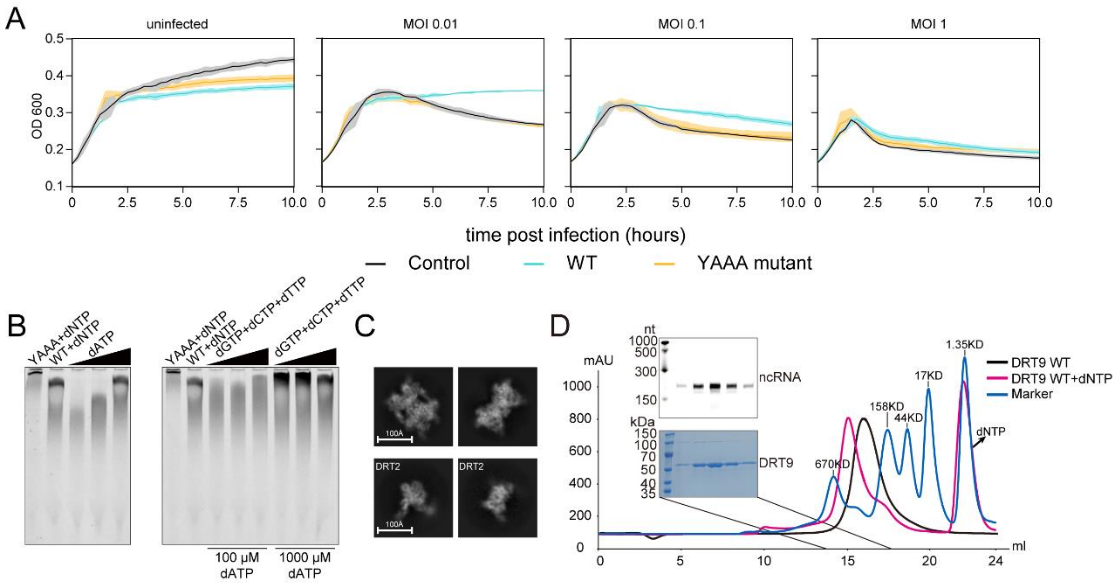

Appendix Figure S1. Biochemical functions of DRT9 system.

(A) Growth curves of BL21-AI cells infected with phage T5 at different multiplicities of infection (MOIs). The lines represent the means and the shades areas the lines show the means ± standard error of three replicates. **(B)** Left panel, in vitro reverse transcription assay supplemented with different concentrations of dATP substrates. Right panel, in vitro reverse transcription assay of DRT9 system added with various concentrations of dGTP+dCTP+dTTP at a certain concentration of dATP. The gels represent three replicates. **(C)** Cryo-EM 2D classification images of DRT2 and DRT9 systems using the same extraction box size (256 pixels). The pixel sizes of DRT2 and DRT9 datasets are 0.85 Å/pixel and 0.95 Å/pixel, respectively. **(D)** Size exclusion chromatograms of DRT9 system. The peak fractions containing DRT9 complex were analyzed by SDS-PAGE and urea-PAGE. The results indicate that DRT9 would undergo an oligomerization transition in the presence of dNTPs. The theoretical molecular weights of the tetrameric and hexameric DRT9 complexes are ~ 450 kDa and 690 kDa, respectively, which are close to the experimental data calculated by the protein standards. The gels are representative of three repeat experiments.

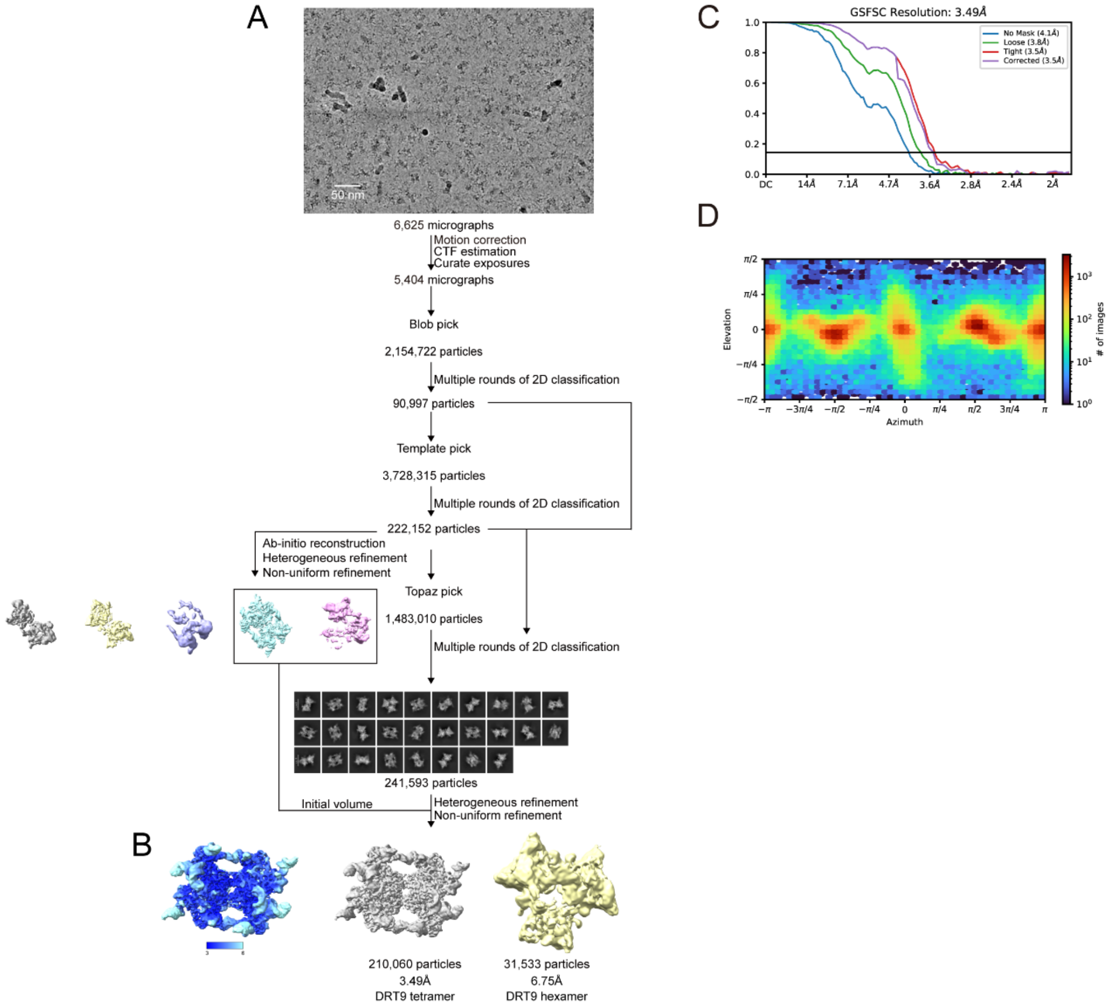

Appendix Figure S2. Cryo-EM data processing of DRT9 tetramer complex.

(A) The flowchart of DRT9 tetramer data processing. **(B)** Local resolution map of DRT9 tetramer. **(C)** FSC curve of DRT9 tetramer. **(D)** Viewing direction distribution of the particles in the DRT9 tetramer map.

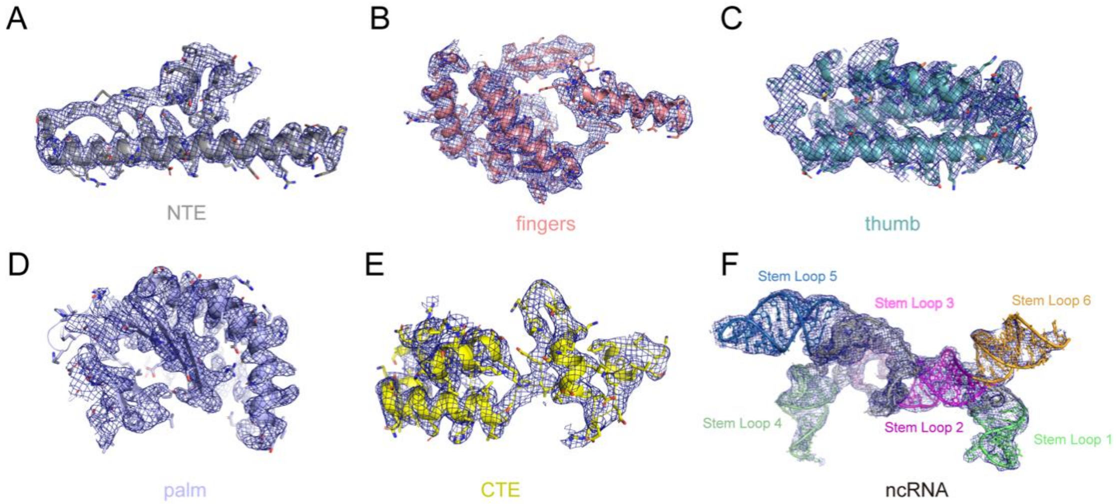

Appendix Figure S3. Structures and corresponding density maps of different DRT9-RT domains and ncRNA. The domain colors are consistent with **Fig. 2A** and distinct stem-loop regions of the ncRNA are represented in varying colors. The corresponding density maps are shown at the same contour level.

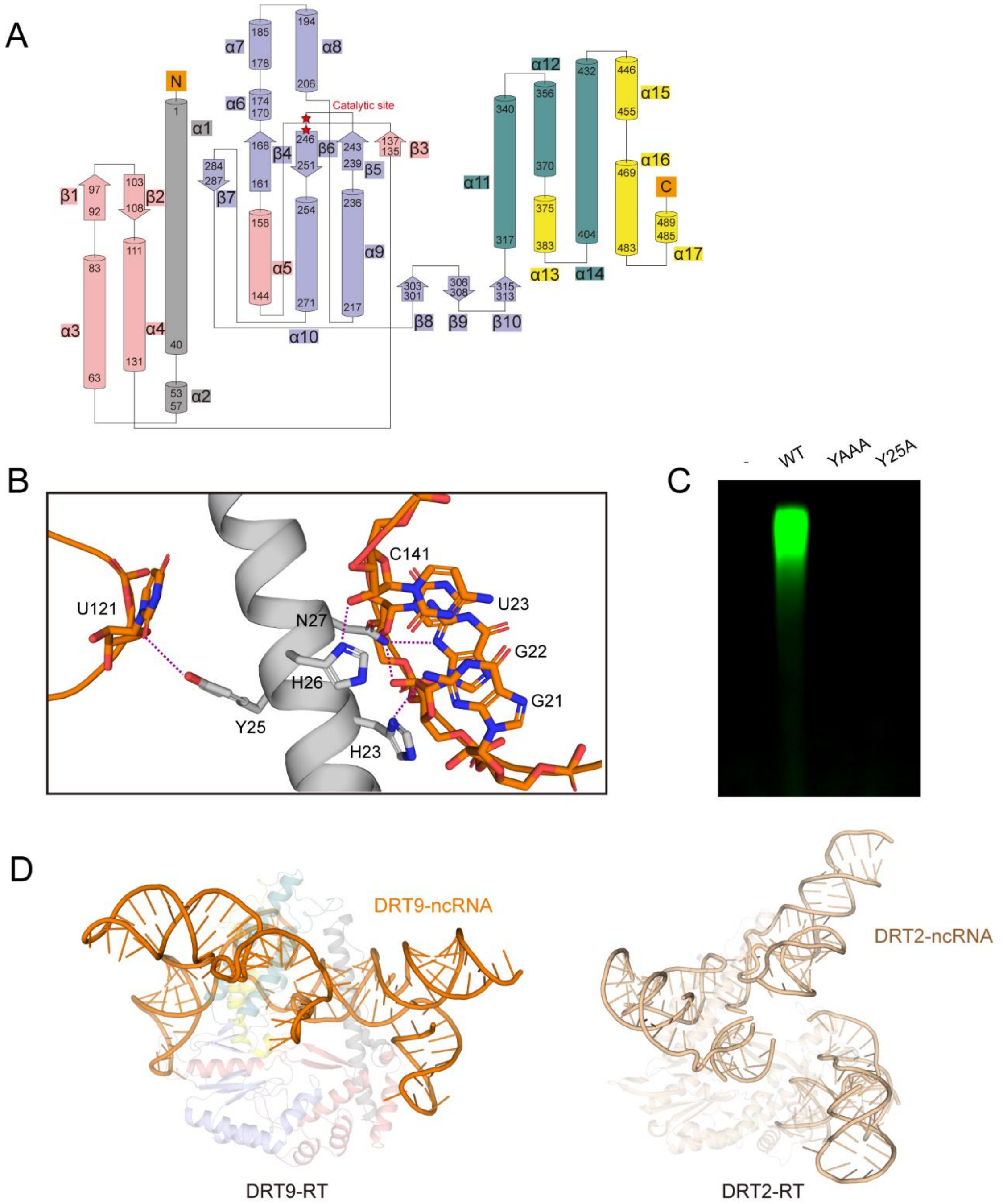

Appendix Figure S4. NTE domain is important to the activation of DRT9 system.

(A) Topological structure of DRT9 protein. The domain color is consistent with **Fig. 2A**. **(B)** Close-up view of interaction between the NTE domain and ncRNA. Key interacting residues and nucleotides are shown as sticks. **(C)** Assay of WT and mutant DRT9 reverse transcriptase activity. The gel represents three independent and replicate experiments. **(D)** Structure comparison between DRT9 ncRNA (orange) and DRT2 ncRNA (wheat).

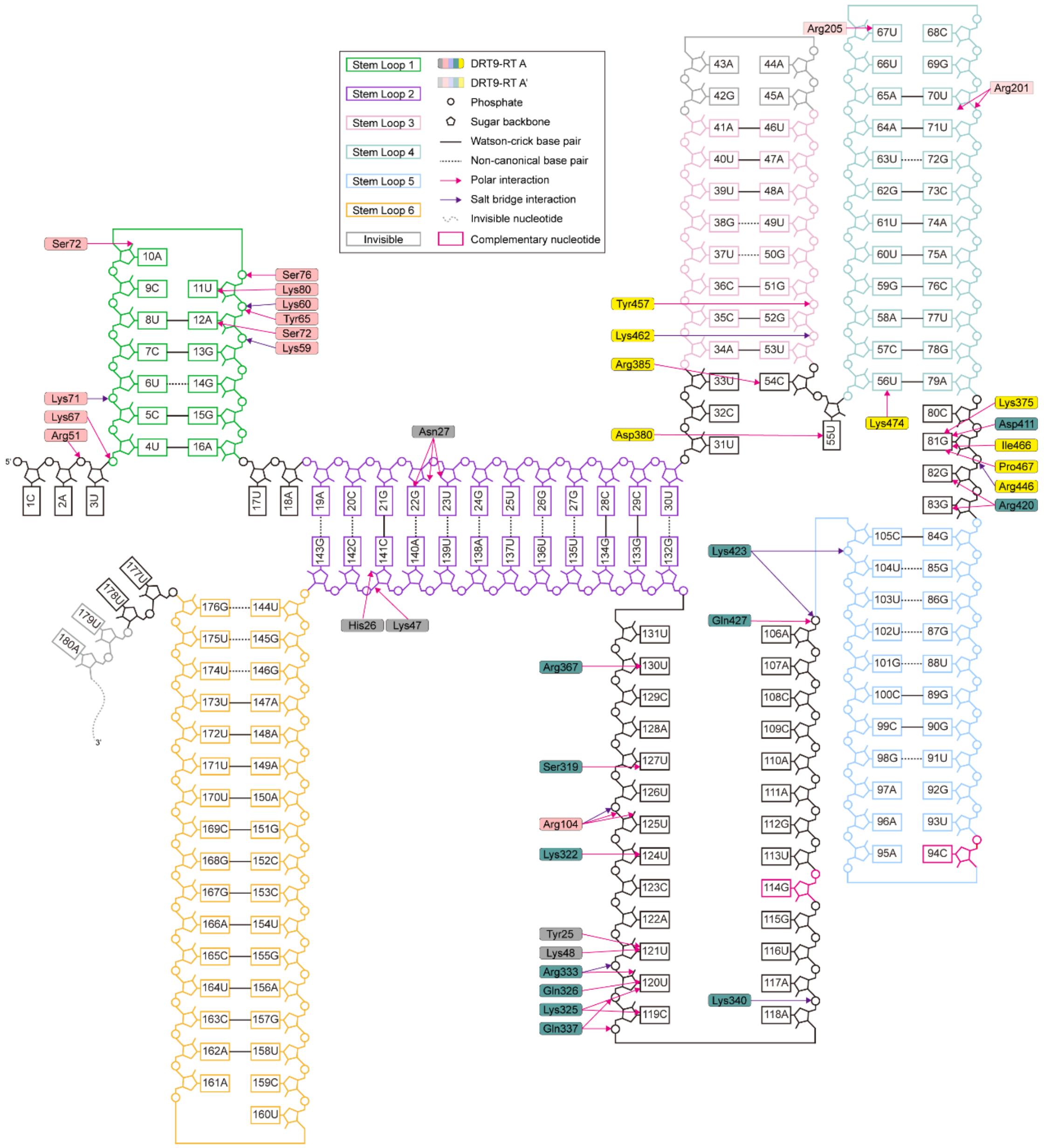

Appendix Figure S5. Interactions between DRT9-RT and the ncRNA.

The same color scheme as in **Fig. 2A**.

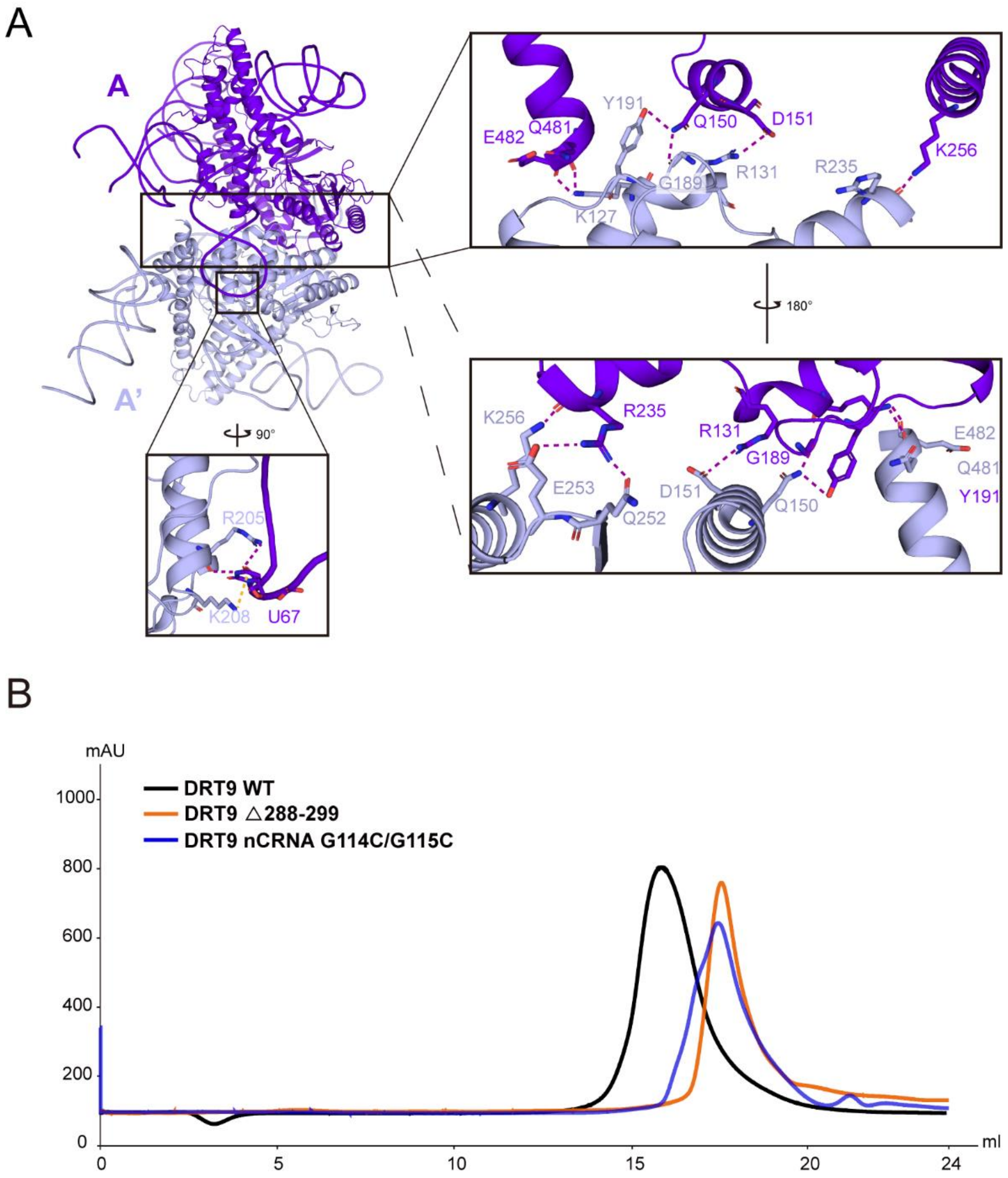

Appendix Figure S6. Key interaction responsible for DRT9 dimer unit formation.
(A) Detailed insights into the intra-unit interactions mediated by the DRT9 dimer. Key residues responsible for the interactions are shown as sticks. The lower panel shows the protein-ncRNA interaction responsible for dimerization. Two basic residues, R205 and K208, are contacted with U67 and shown as sticks. **(B)** Superimposed size exclusion chromatography curves of DRT9 variants.

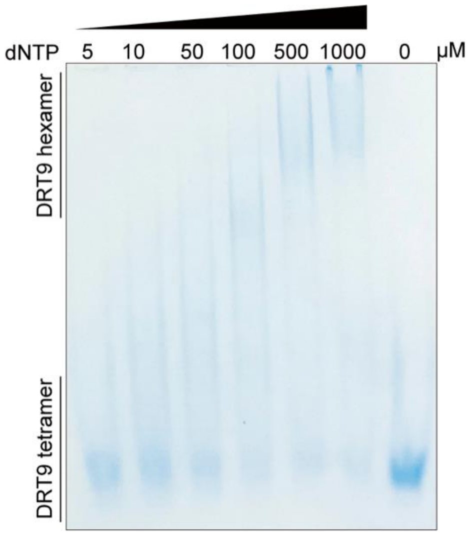

Appendix Figure S7. dNTPs induce oligomerization transition of DRT9.

Native gel analysis of DRT9 in different dNTP. The gel represents three replicate experiments.

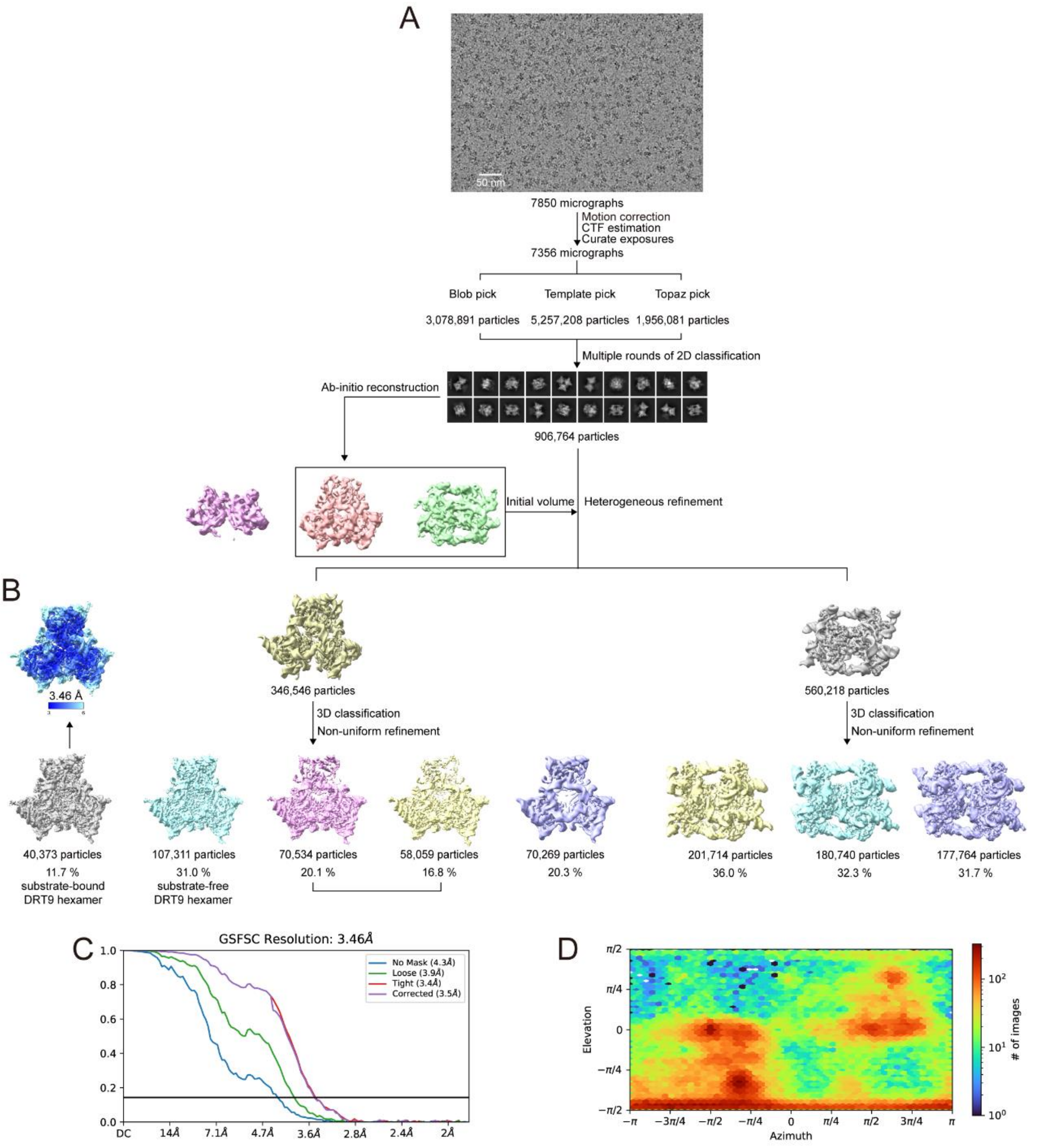

Appendix Figure S8. Cryo-EM data processing of DRT9 hexamer complex.

(A) The flowchart of DRT9 hexamer data processing. **(B)** Local resolution maps of substrate-bound DRT9 hexamer. The corresponding bar is next to it. **(C)** FSC curve of substrate-bound DRT9 hexamer. **(D)** Viewing direction distribution of the particles in the substrate-bound DRT9 hexamer map.

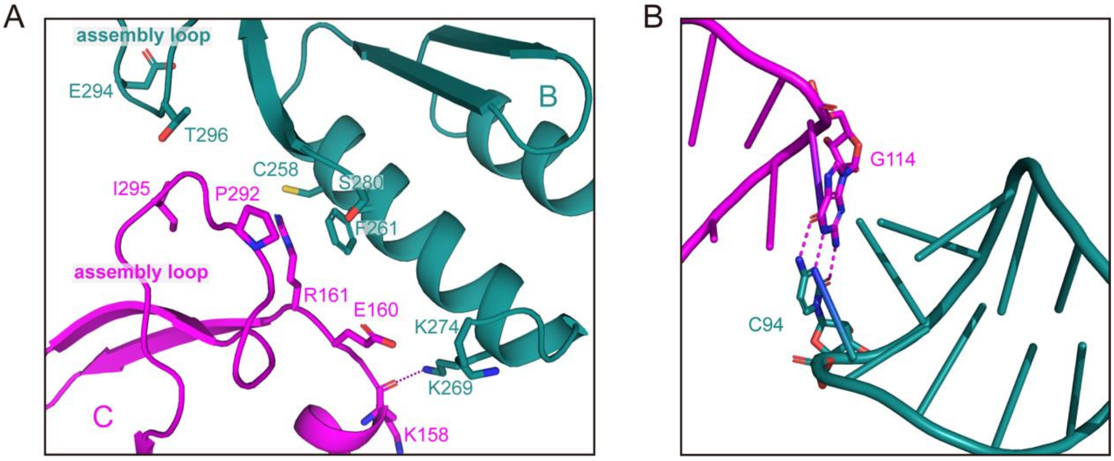

Appendix Figure S9. The inter-unit interaction of DRT9 hexamer.

(A) Focused view of inter-unit interface of DRT9 hexamer. The key residues responsible for protein-protein interactions are shown as sticks. **(B)** The C94-G114 base pair is crucial for inter-unit interaction of DRT9 hexamer.

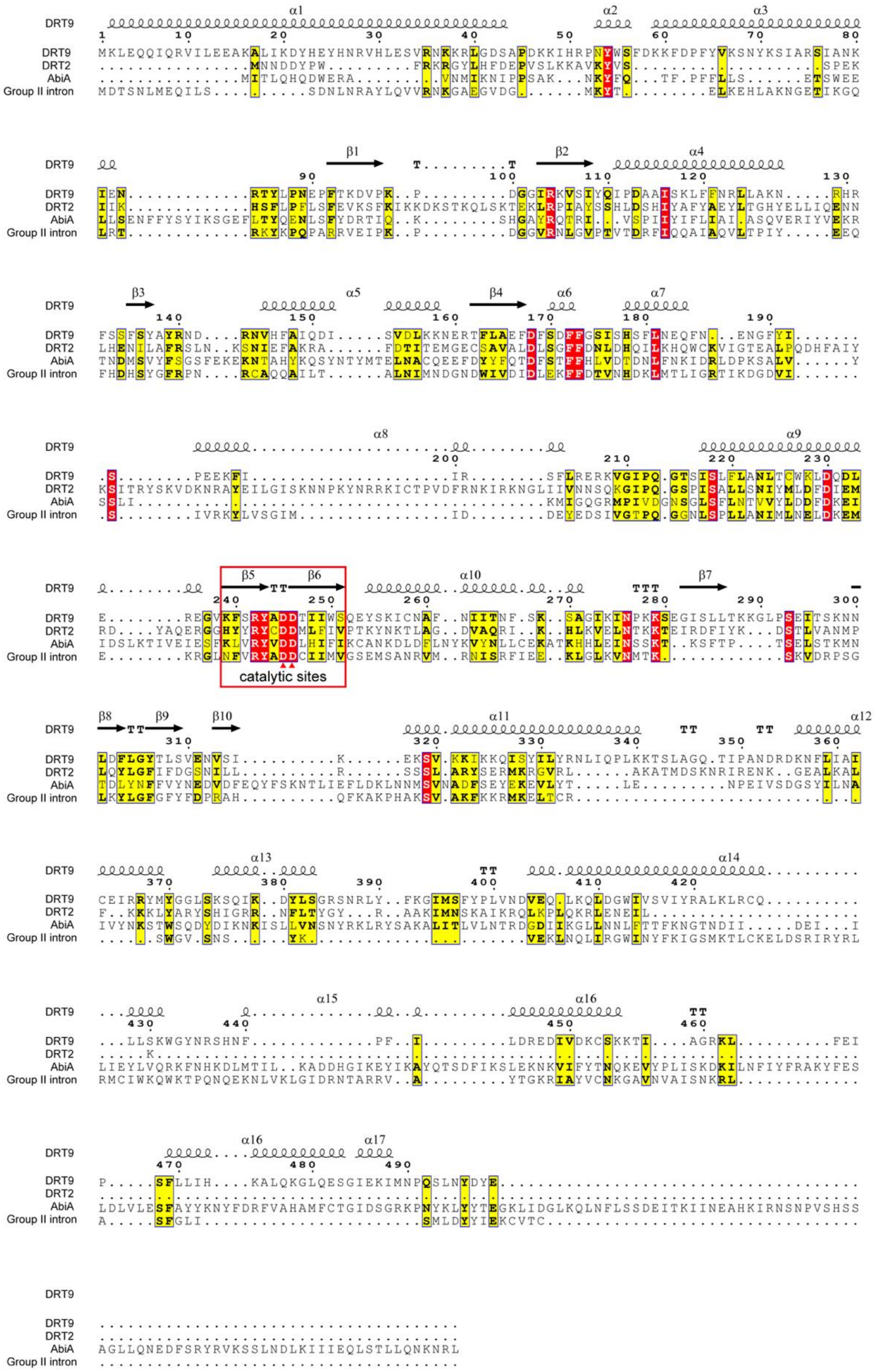

Appendix Figure S10. Multiple sequence alignment of DRT9 and its homologs.

The conserved catalytic residues of these reverse transcriptases are framed by a red square and marked with red triangles.

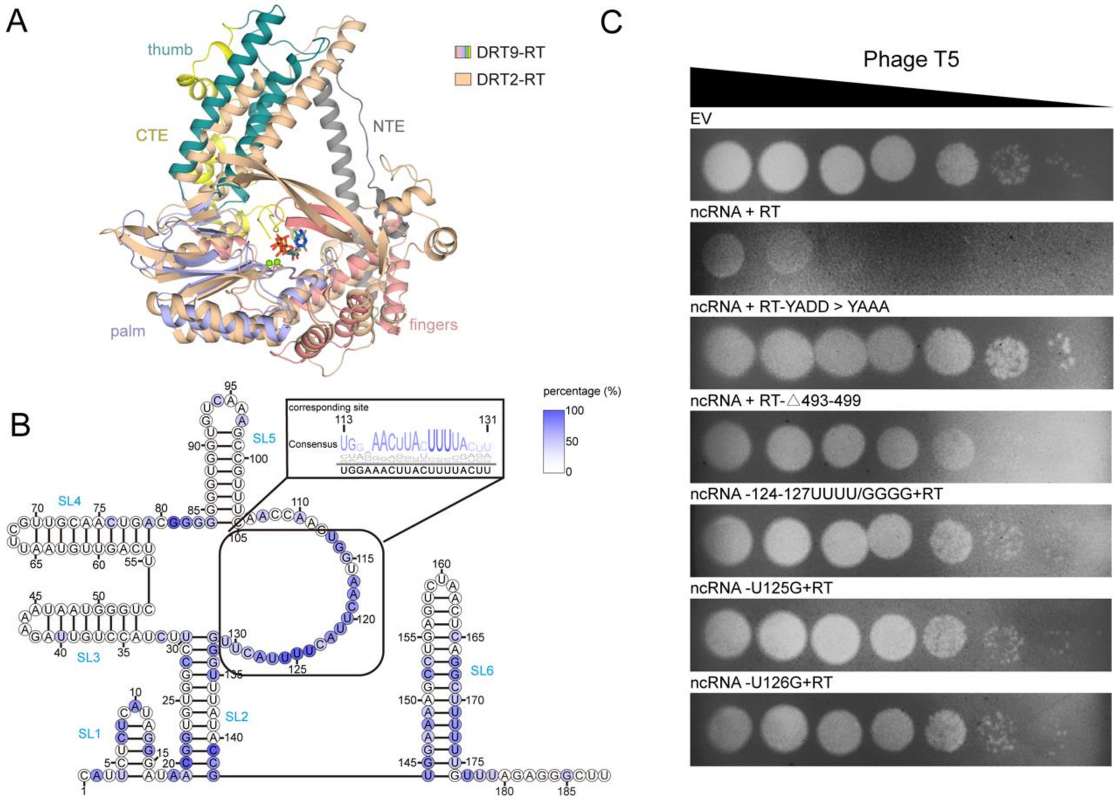

Appendix Figure S11. The conserved nucleotides are important for the defense activity of DRT9 system.

(A) Structural superposition of DRT2-RT and DRT9-RT. The DRT2-RT protein is colored in wheat. The DRT9-RT protein is colored in the same scheme as **Fig. 2A**. **(B)** Multiple sequence alignment of predicted ncRNA sequences of DRT9 orthologs. The DRT9 ncRNA is colored based on percentage identity. The inserted panel represents conserved nucleotide sequences. **(C)** Plaque assay of BL21-AI cells carrying empty vector (EV) and DRT9 system variants. RT-YADD > YAAA is a variant of DRT9-RT with the catalytic sites mutated. The images represent three repeat experiments.

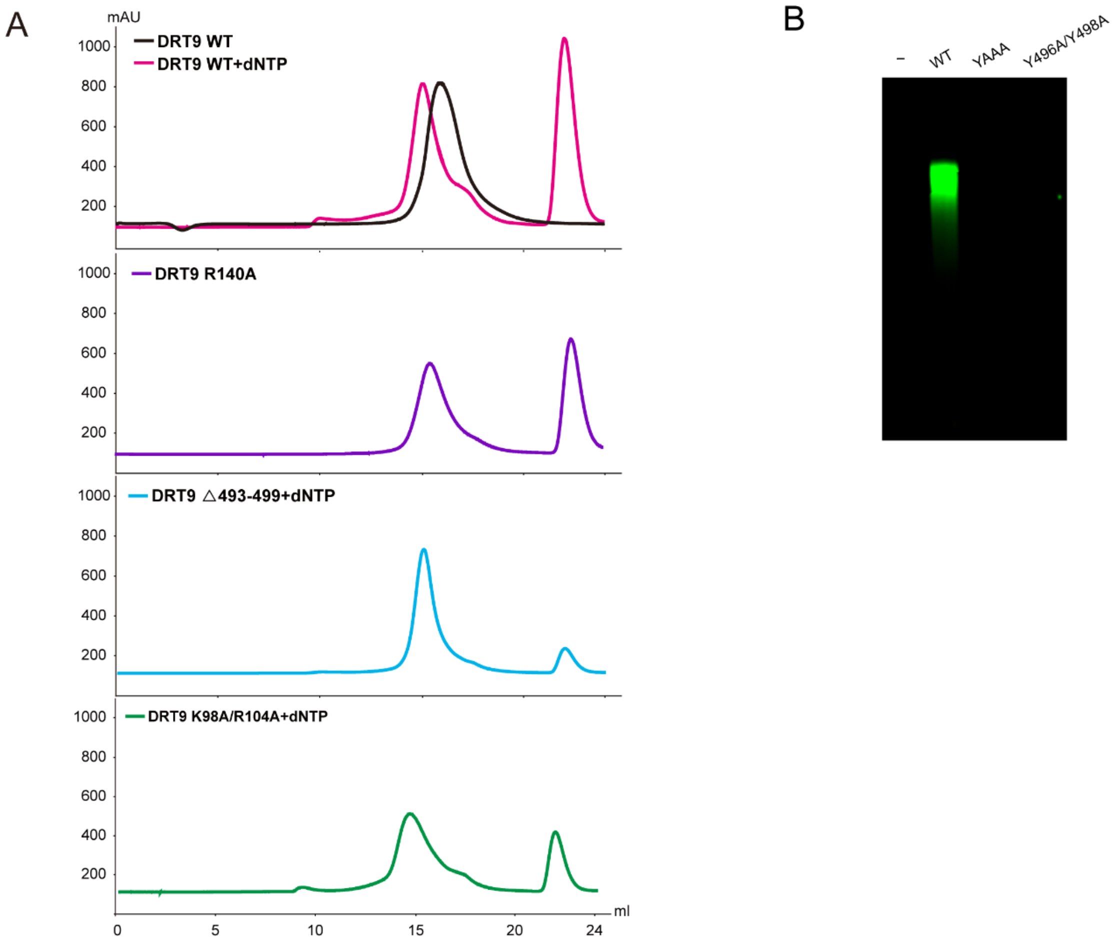

Appendix Figure S12. Analysis of oligomerization and reverse transcriptase activity in DRT9 mutants.

(A) Size exclusion chromatography profile of DRT9 WT and loss-of-function mutants. The results suggest that these loss-of-function mutants do not alter the oligomerization status. **(B)** The Y494/Y498A mutant exhibits no reverse transcriptase activity. The gel is representative of three replicates.

Appendix Table S1. Cryo-EM data collection and refinement statistics

	DRT9 tetramer complex (EMD-65143) (PDB 9VKU)	substrate-bound DRT9 hexamer complex (EMD-65181) (PDB 9VMA)
**Data collection and processing**		
Magnification	50,000×	50,000×
Voltage (kV)	300	300
Electron exposure ($e^-/Å^2$)	40	40
Defocus range (μm)	-0.5 to -2.5	-0.5 to -2.5
Pixel size (Å)	0.95	0.95
Symmetry imposed	C1	C1
Initial particle images (no.)	241,593	906,764
Final particle images (no.)	210,060	40,373
Map resolution (Å)	3.49	3.46
FSC threshold	0.143	0.143
Map resolution range (Å)	2.0 to 4.0	2.0 to 4.0
**Refinement**		
Initial model used	AlphaFold	AlphaFold
Model resolution (Å)	3.6	3.3
FSC threshold	0.5	0.5
Model resolution range (Å)	3.4 to 3.8	3.4 to 4.1
Map sharpening *B* factor ($Å^2$)	-109.8	-58.4
Model composition		
Non-hydrogen atoms	30120	45606
Protein residues	1977	2976
Nucleotides	652	987
B factors ($Å^2$)		
Protein	137.68	107.84
Nucleotides	213.70	181.19
R.m.s. deviations		
Bond lengths (Å)	0.004	0.006
Bond angles (°)	0.844	0.951
Validation		
MolProbity score	1.51	1.85
Clashscore	6.61	10.43
Poor rotamers (%)	0.39	2.56
Ramachandran plot		
Favored (%)	97.21	98.18
Allowed (%)	2.79	1.75
Disallowed (%)	0	0.07
