# Supplementary figures and images for: Non-coding RNA mediates the defense-associated reverse transcriptase (DRT) anti-phage oligomerization transition
Source: EMBO J. 2025 Aug 20;44(19):5429–42. doi: 10.1038/s44318-025-00544-8 (PMC12489045; doi:10.1038/s44318-025-00544-8)

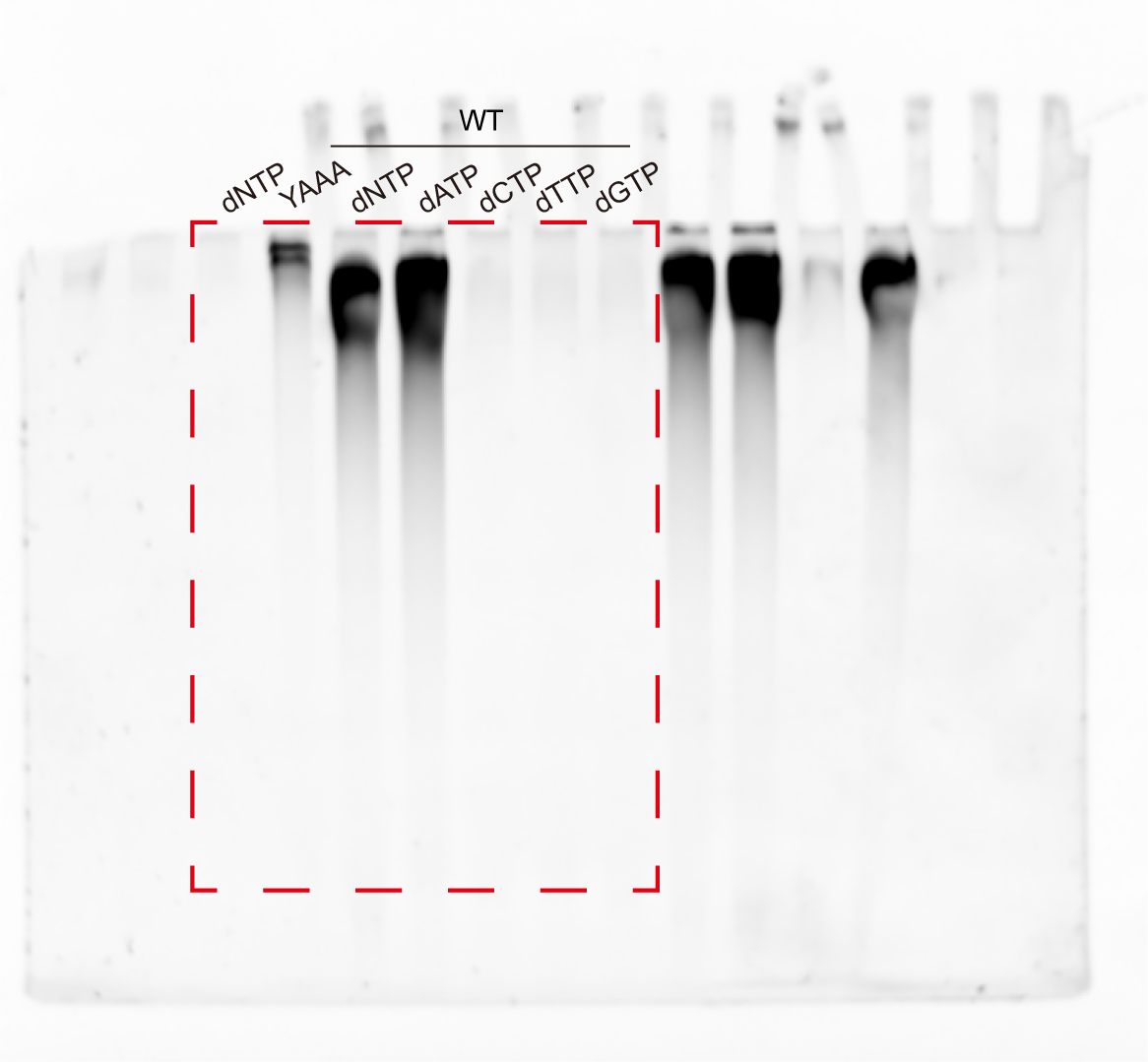

Supplement: Supplementary file 3 — Source data Fig. 1 [file 44318_2025_544_MOESM3_ESM.zip › Figure 1/1F/1F.tif]

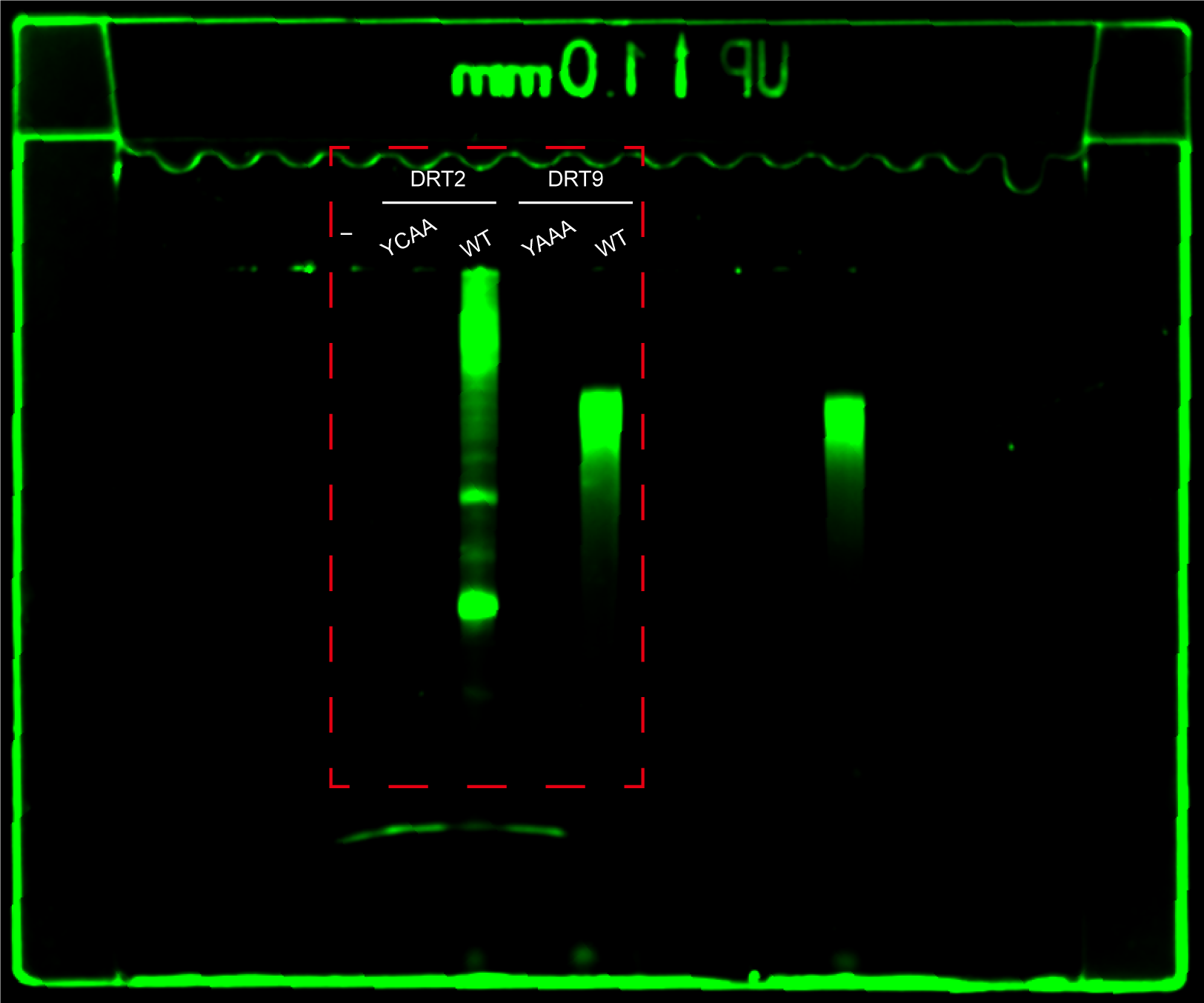

Supplement: Supplementary file 3 — Source data Fig. 1 [file 44318_2025_544_MOESM3_ESM.zip › Figure 1/1D/1D.tif]

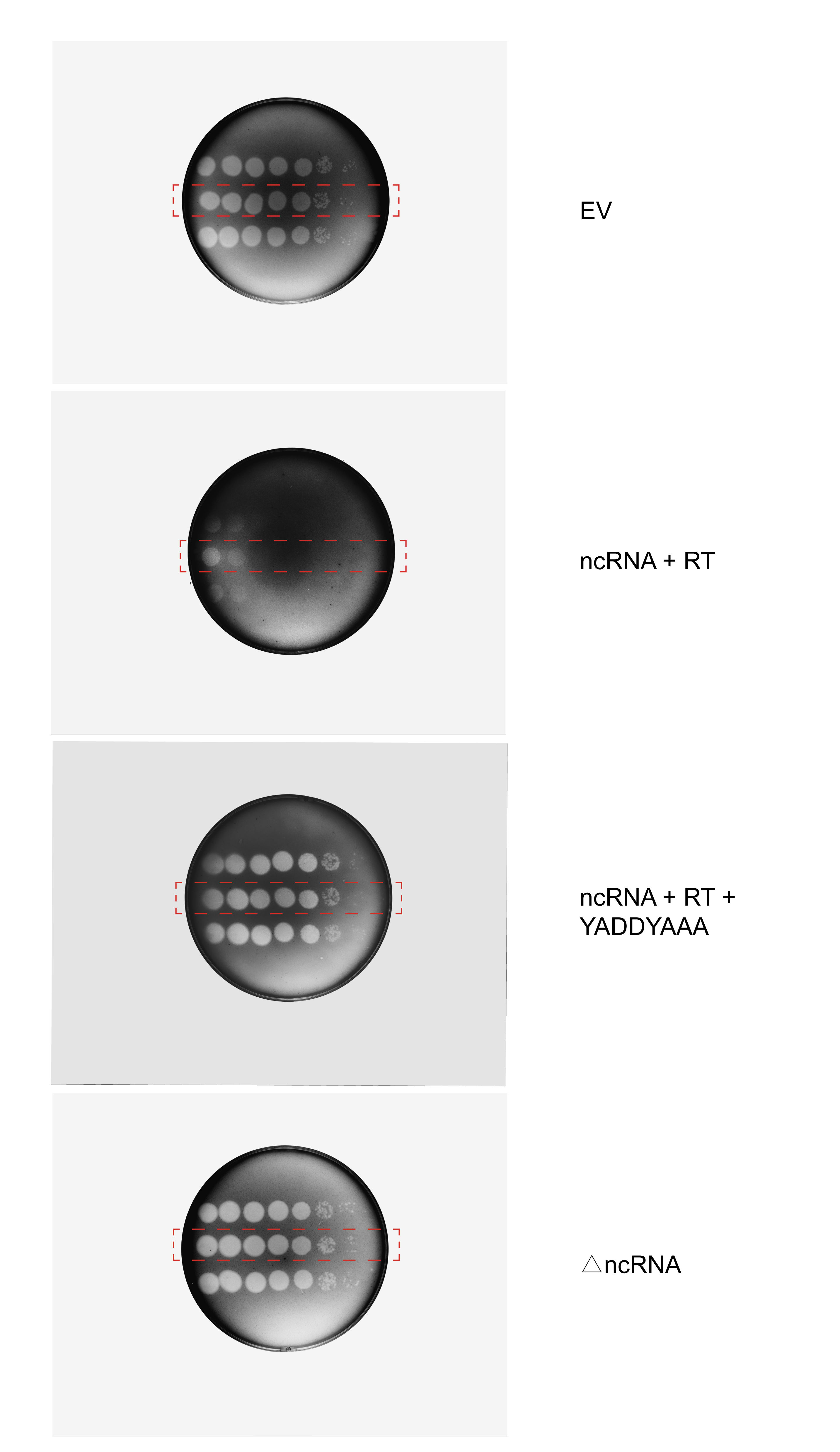

Supplement: Supplementary file 3 — Source data Fig. 1 [file 44318_2025_544_MOESM3_ESM.zip › Figure 1/1C/1C.tif]

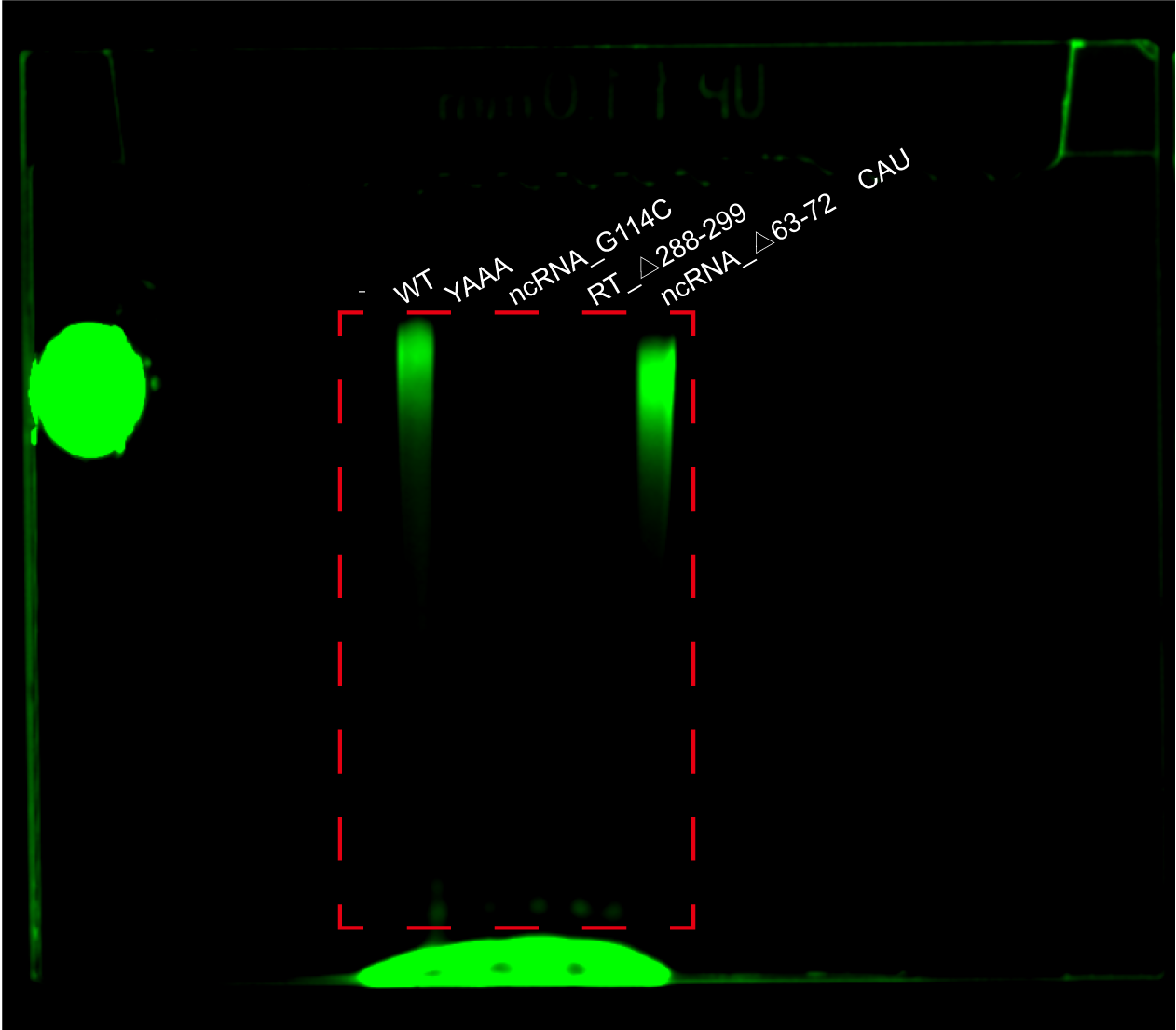

Supplement: Supplementary file 4 — Source data Fig. 3 [file 44318_2025_544_MOESM4_ESM.zip › Figure 3/3E/3E.tif]

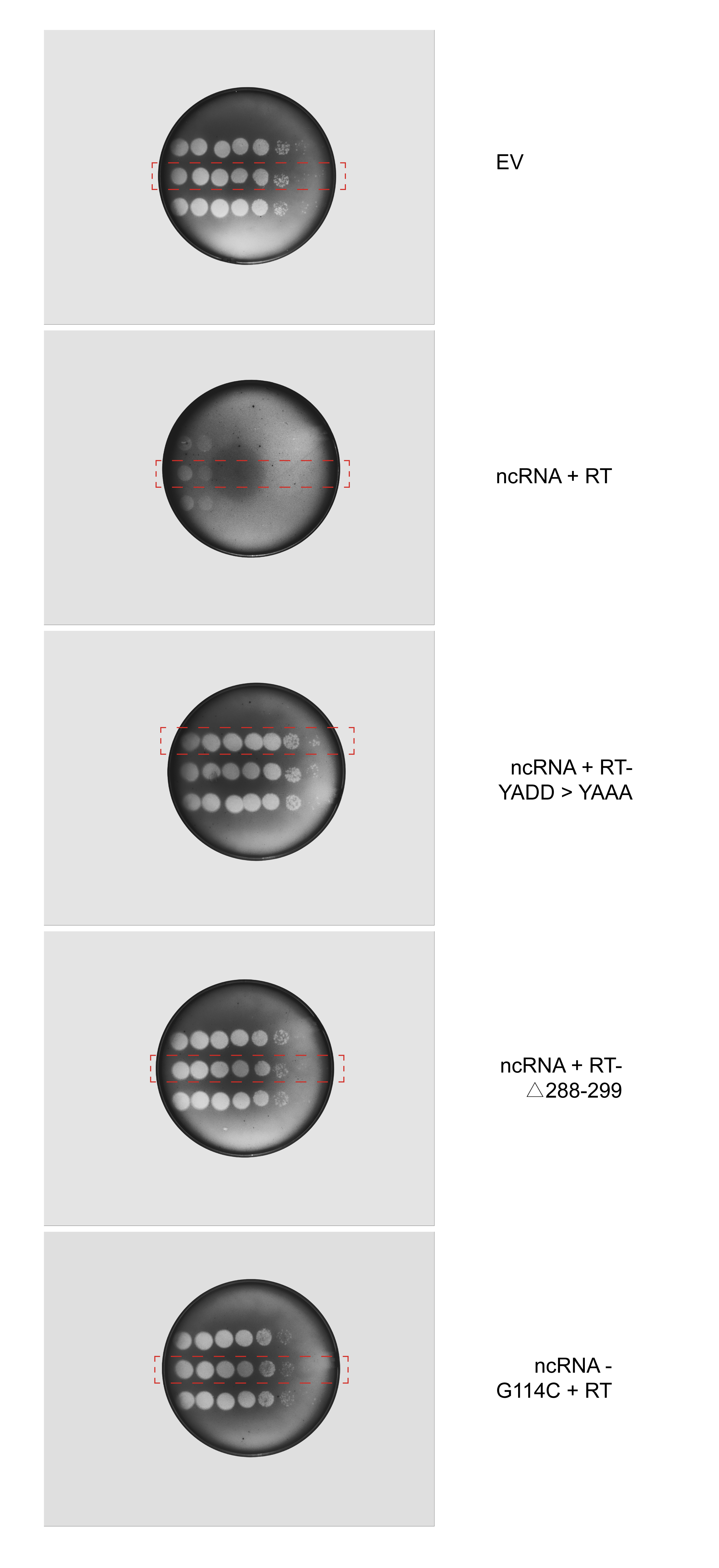

Supplement: Supplementary file 4 — Source data Fig. 3 [file 44318_2025_544_MOESM4_ESM.zip › Figure 3/3F/3F.tif]

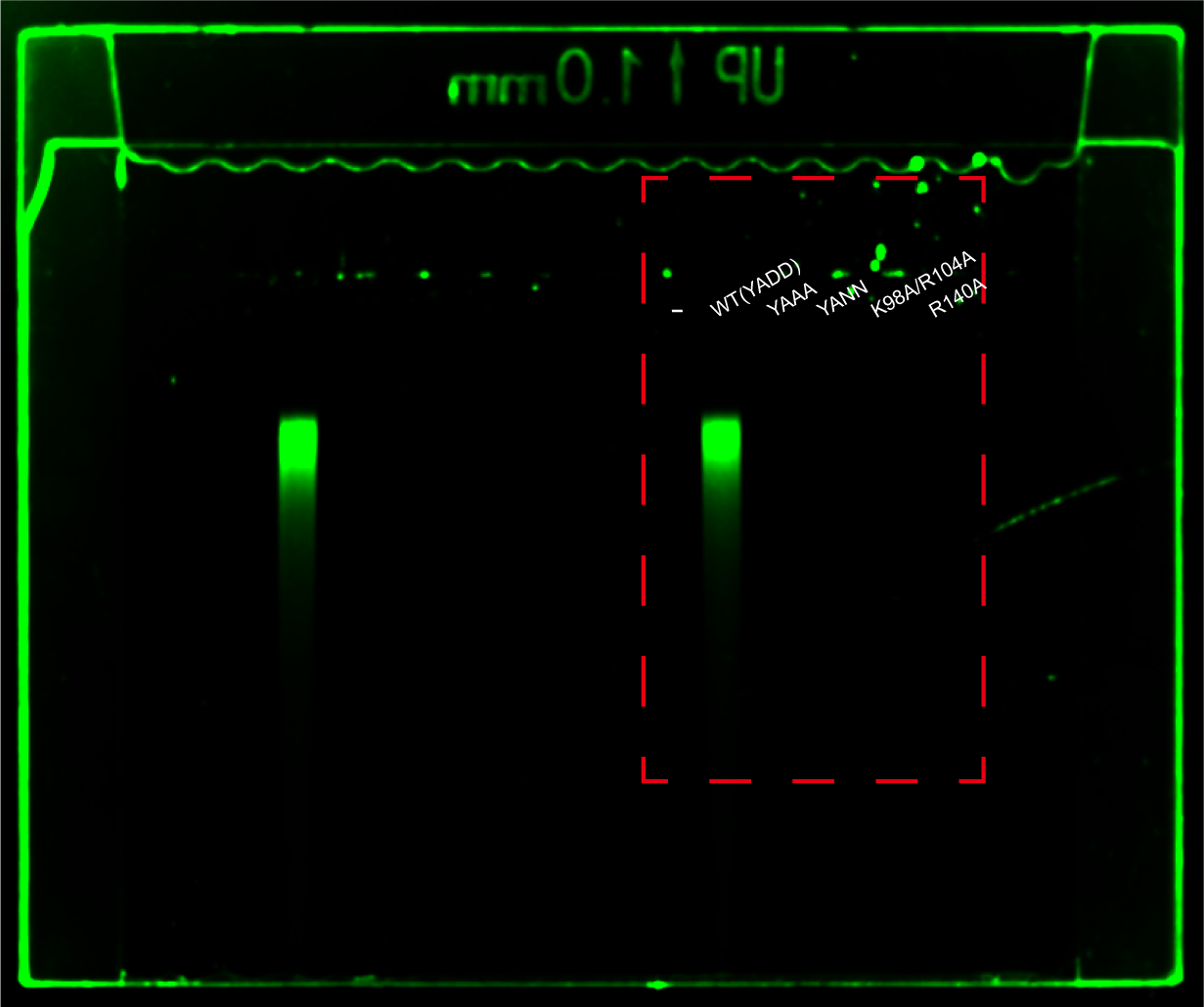

Supplement: Supplementary file 5 — Source data Fig. 5 [file 44318_2025_544_MOESM5_ESM.zip › Figure 5/5F/5F.tif]

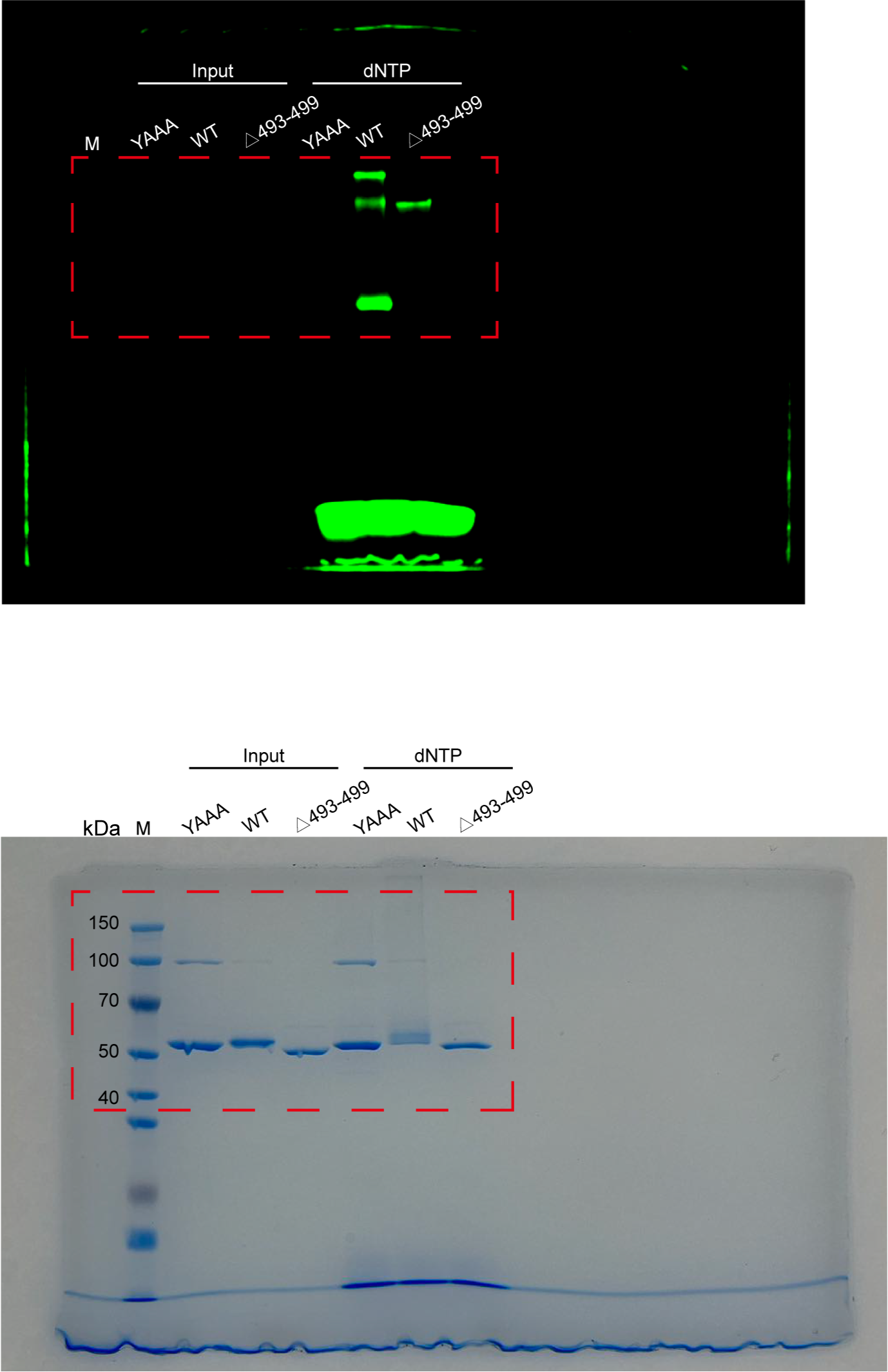

Supplement: Supplementary file 6 — Source data Fig. 6 [file 44318_2025_544_MOESM6_ESM.zip › Figure 6/6D/6D.tif]

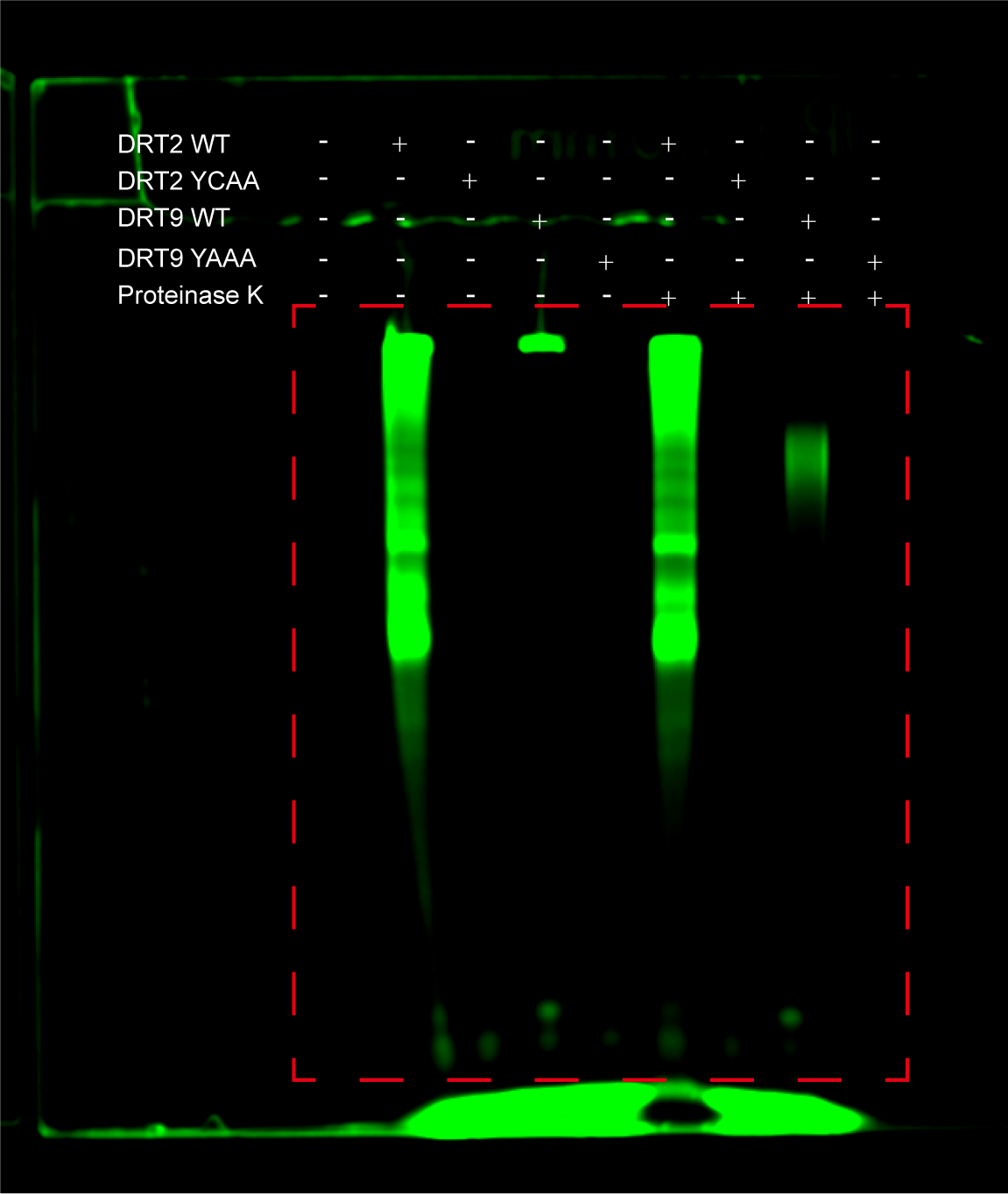

Supplement: Supplementary file 6 — Source data Fig. 6 [file 44318_2025_544_MOESM6_ESM.zip › Figure 6/6C/6C.tif]
